# Early prediction of sudden cardiac death risk with Nested LSTM based on electrocardiogram sequential features

**DOI:** 10.1186/s12911-024-02493-4

**Published:** 2024-04-10

**Authors:** Ke Wang, Kai Zhang, Banteng Liu, Wei Chen, Meng Han

**Affiliations:** 1 College of Information Science and Technology, Zhejiang Shuren University, Hanzhou, 310015 China; 2 Comprehensive Technical Service Center of Wenzhou Customs, Wenzhou, 325299 China; 3 Zhejiang University, Hanzhou, 310058 China; 4 Binjiang Institute of Zhejiang University, Hanzhou, 310053 China

**Keywords:** Time series prediction, Nested LSTM, ECG signal, Sudden cardiac death

## Abstract

Electrocardiogram (ECG) signals are very important for heart disease diagnosis. In this paper, a novel early prediction method based on Nested Long Short-Term Memory (Nested LSTM) is developed for sudden cardiac death risk detection. First, wavelet denoising and normalization techniques are utilized for reliable reconstruction of ECG signals from extreme noise conditions. Then, a nested LSTM structure is adopted, which can guide the memory forgetting and memory selection of ECG signals, so as to improve the data processing ability and prediction accuracy of ECG signals. To demonstrate the effectiveness of the proposed method, four different models with different signal prediction techniques are used for comparison. The extensive experimental results show that this method can realize an accurate prediction of the cardiac beat’s starting point and track the trend of ECG signals effectively. This study holds significant value for timely intervention for patients at risk of sudden cardiac death.

## Introduction

Sudden cardiac death is defined as a death occurring usually within an hour of onset of symptoms, arising from an underlying cardiac disease. Sudden cardiac death is a complication of a number of cardiovascular diseases and is often unexpected [1, 2]. Clinical manifestations may include chest pain, shortness of breath, fatigue, weakness, persistent angina pectoris, arrhythmia, etc. [3]. At present, there are limited effective methods to predict the occurrence of SCD in individuals without prior cardiac issues. As a simple, easy-to-use, reliable ECG analysis tool, the electrocardiogram (ECG) provides abundant information for the diagnosis and treatment of cardiovascular disease. Based on ECG signals, abnormal and significant fluctuations can be detected in patients before the onset of SCD [4, 5]. For example, Ventricular Fibrillation (VF) is an important manifestation of sudden cardiac death, and the trend of VF can be obtained by monitoring ECG signals [6, 7]. However, ECG is a weak signal with strong nonlinearity, non-stationarity, and randomness, which affect the final diagnostic results. Therefore, accurate prediction of ECG signals plays a pivotal role in the early detection and prevention of Sudden Cardiac Death [8, 9].

At present, the traditional machine learning models are commonly applied for ECG prediction. These models make forecasting based on historical data, such as classification model and regression model [10–13]. For example, Liu et al. [14] developed a cardiac arrest classification model utilizing wavelet transform and the AdaBoost algorithm. This model effectively distinguishes cardiac arrest from ECG signals and predicts its occurrence with an impressive accuracy of 97.56% within 5 minutes before the event. Ebrahimzadeh et al. [15] employed a Multi-Layer Perceptron (MLP) to classify abnormal ECG signals, with the aim of predicting Sudden Cardiac Death (SCD). Their model demonstrates increasing prediction accuracy as it approaches the critical point of sudden death. Sengupta et al. [16] utilized Random Forest, Least Squares Discriminant, and Support Vector Machine in the classification of 12-lead ECG signals to predict abnormal myocardial relaxation and assess the likelihood of SCD. Hou et al. [17] presented a novel deep learning-based algorithm that combined an LSTM-based auto-encoder (LSTM-AE) network with support vector machine (SVM) for ECG arrhythmias classification. This method exhibits high accuracy, sensitivity, and specificity in classifying various heartbeat types, showcasing its potential for ECG arrhythmia classification. Kaya et al. [18] proposed an innovative approach that combines angle transform (AT) and LSTM for the automatic identification of congestive heart failure (CHF) and arrhythmia (ARR) using ECG signals. However, most of these methods achieve classification based on patients’ ECG signals and those of healthy individuals, which struggles to address dynamic system modeling problems related to time [19, 20].

With the rapid development of artificial intelligence, neural networks has been broadly applied in signal processing and achieves excellent performance. Jin et al. [21] proposed a regression model based on the Regularized Extreme Learning Machine (RELM) to predict ECG signals by analyzing the correlation between ECG and human gait. Zheng et al. [22] employed a traditional Echo State Network (ESN), a type of Recurrent Neural Network, to forecast ECG signals, yielding superior results compared to conventional regression machine learning techniques. Wang et al. [23] devised a cardiac beat prediction algorithm for SCD based on ECG, establishing a time series prediction model for dynamic human ECG signals to accurately anticipate ECG signal patterns. Sakib et al. [24] employed a Reservoir Computing (RC)-based ESN method for magnetocardiography (MCG) monitoring and helped to detect cardiac activity.

In recent years, some powerful sequence models have been proposed to assist with ECG analysis with their advantage of exploring time-frequency based features [25–30]. As one of the most commonly used sequence model, the Long Short-Term Memory (LSTM) network has been proven to be effective to track information over extended periods [31, 32]. For instance, Liu et al. [33] employed LSTM to predict influenza trends and achieved better results than linear models. Balci et al. [34] presented a hybrid Attention-based LSTM-XGBoost algorithm for detecting atrial fibrillation (AF) in long-recorded ECG data. Combined with preprocessing techniques, this method achieves a high accuracy, offering a reliable support system for clinicians and facilitating data tracking in long ECG record reviews.

However, traditional LSTM networks exhibit weak robustness and low prediction accuracy in complex tasks, as the memory cells store memories unrelated to the current time step [35, 36]. To address this issue, we propose an integrated approach combining data preprocessing and prediction model construction to predict ECG signal trends in advance. Firstly, data prepocessing is performed on the original ECG signals, including wavelet denoising, normalization, and phase space reconstruction. Then, the Nested LSTM model is utilized for signal prediction. At this step, an inner LSTM unit is adopted, which can guide the memory forgetting and memory selection of ECG signals, so as to improve the data processing ability and prediction accuracy of ECG signals.

This paper is organized as follows. In Introduction section, a brief introduction of the existing ECG signal prediction methods is made. A detailed algorithm and description of the proposed methods are presented in Theory and calculation section. Experiment and results section provides implementation details of the experiments. The effectiveness and superiority of the proposed method are verified through experiments and results analysis. Conclusion section summarizes this paper.

## Theory and calculation

### Model construction

**Fig. 1:**
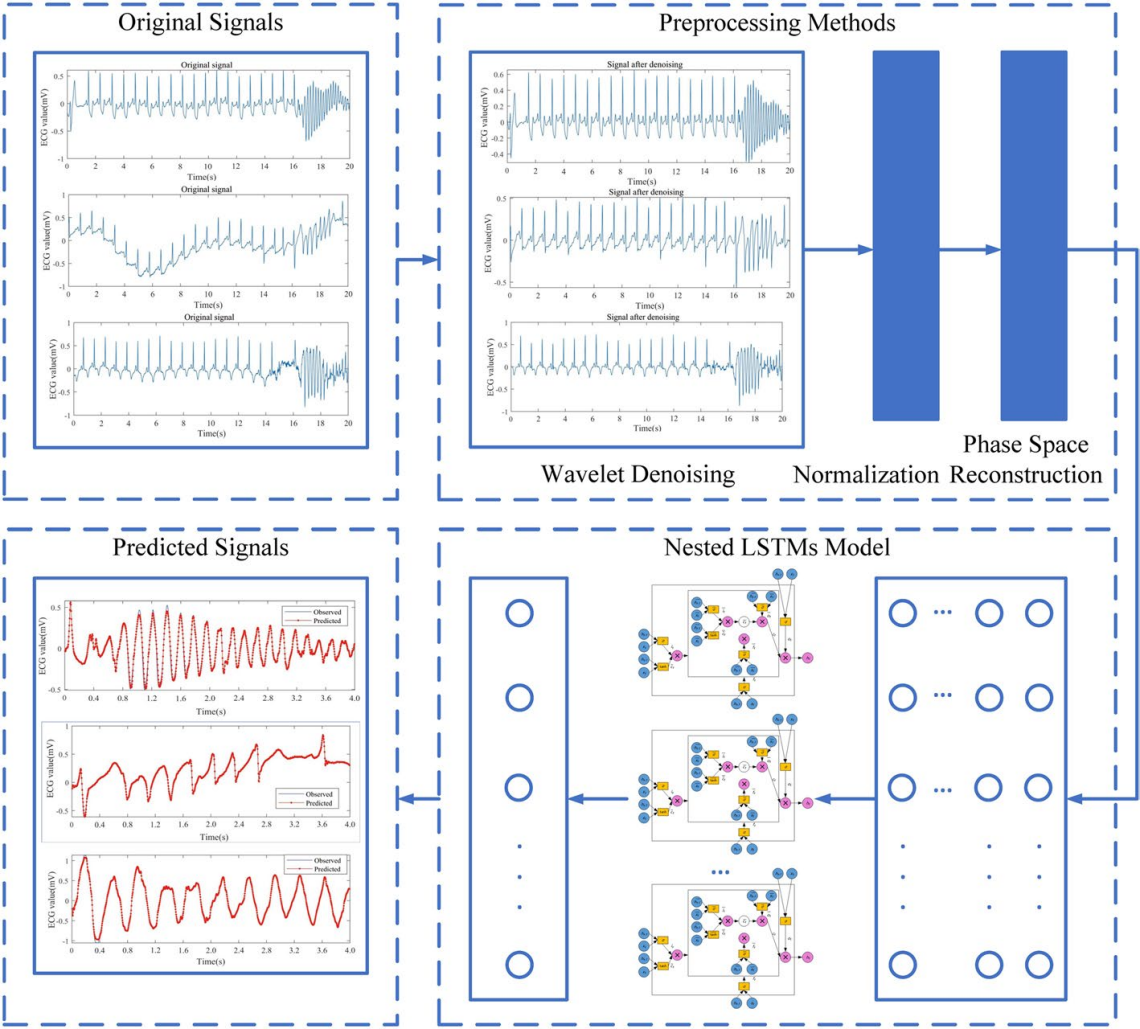
Model construction framework diagram

The model construction proposed in this paper is presented in Fig. 1, including the data preprocessing strategy, the prediction model Nested LSTM and model evaluation.

#### Preprocessing methods

Considering the uncertainty and complexity of ECG signals, it would be a difficult to capture the trend of the data directly. Thus, the data preprocessing strategy is adopted to ensure the quality of data, after which some unwanted noise are removed from the ECG signals. Therefore, we employ a data preprocessing strategy to ensure data quality and remove unwanted noise from the ECG signals. The preprocessing methods include signal denoising, normalization, and phase space reconstruction.

##### Signals denoising

Due to the movement of human limbs, breathing, electromagnetic interference of the surrounding environment, ECG signals are accompanied by a lot of noise, including baseline drift, power frequency interference, electromyographic interference, and motion artifacts, which could have a certain impact on the prediction results. The frequency ranges and signal energy ranges of the four types of noise are as follows: (1) Baseline drift noise: the noise frequency is less than 5Hz; the energy range is between 0.01 Hz and 1 Hz. (2) Power frequency interference noise: the noise frequency range is 50 Hz or 60 Hz; the energy range is concentrated in frequency components near the power frequency. (3) Myoelectric interference noise: The noise frequency range is in the range of 5HZ 2000Hz. The energy range is between 10 Hz and 500 Hz, depending on the frequency of muscle activity. (4) Motion artifact noise: Noise frequency range is in the range of 3Hz 14Hz, depending on the frequency and amplitude of motion. The energy range is in the range of 5Hz 10Hz, reflecting the frequency components caused by the subject’s movement.

**Fig. 2:**
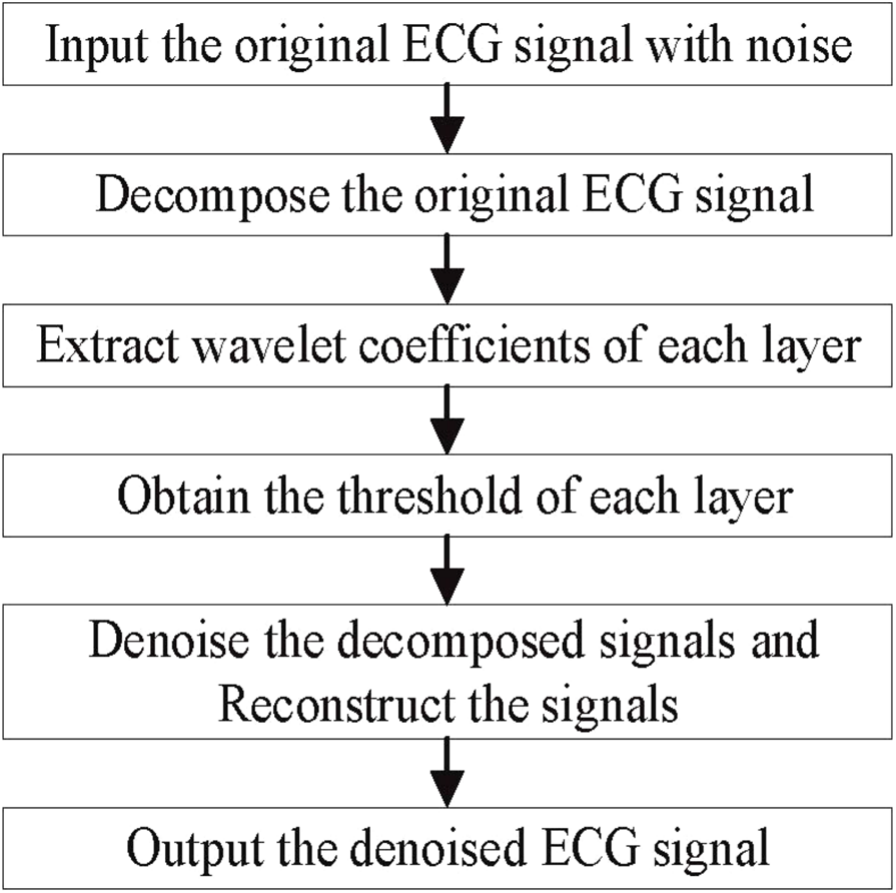
Wavelet denoising flowchart

Therefore, it is necessary to denoise the ECG signals. Wavelet denoising method has the characteristics of multi-resolution analysis and has the ability to characterize the local characteristics of signals in both time and frequency domains. It is very suitable for analyzing nonstationary signals such as ECG signals and extracting the local characteristics of signals. Therefore, the wavelet denoising method is used to denoise the ECG signals in this paper. Its process is shown in Fig. 2. The specific steps are as follows:

- Input the original ECG signals containing noise;
- Decompose the original ECG signals in 7 layers through the wavelet transform in the wavelet denoising method, and the wavelet function selects DB6;
- Extract wavelet coefficients of each layer, including approximation coefficients and detail coefficients;
- Obtain the threshold of each layer by using unbiased likelihood estimation; that is, give a threshold *L* for each layer, calculate its likelihood estimate, and then minimize the likelihood of *L* to obtain the threshold of each layer. The details of determining the threshold are as follows:
  - Step 1: After squaring the wavelet coefficients of each resolution level, arrange them in order from small to large, and obtain the vector $P=[P_1,P_1,...,P_N]$, where *N* represents the length of the wavelet coefficient.
  - Step 2: Calculate the risk vector *R* based on the vector *P*, and find the smallest $R_i$ in the risk vector as the risk value. The formula is as follows:
    1 $$\begin{aligned} R=[R_1,R_1,...,R_N] \end{aligned}$$
    2 $$\begin{aligned} R_i=[N-2*i+(N-i)*P_i+sum(P_k)]/N \end{aligned}$$
  - Step 3: The threshold value *L* is calculated from the square of the wavelet coefficient $P_i$ corresponding to the risk value $R_i$:
    3 $$\begin{aligned} L=median(abs(W(j-1,k)))/0,6745*sqrt(P_i) \end{aligned}$$
- Denoise the decomposed 7-layer signals according to the selected threshold; if ${X_i}$ is the ECG signals of the *i*-th layer after denoising, and ${d_i}$ is the ECG signals of the *i*-th layer before denoising, the denoising method of each layer as follows:
  4 $$\begin{aligned} {X_i} = \left\{ \begin{array}{ll} {d_i},&{} {X_i} \ge {L_{i}} \\ 0,&{} {X_i} < {L_i} \end{array}\right. i = 1,2, \cdots ,{7} \end{aligned}$$
- Reconstruct the 7-layer signals through inverse wavelet transform; the reconstructed ECG signals is:
  5 $$\begin{aligned} X = {X_1} + {X_2} + \cdots + {X_{7}} \end{aligned}$$
  We adopted the method of wavelet reconstruction after wavelet transform, that is, using the inverse wavelet transform method. The steps are as follows:
  - Step 1: For the highest level of detail coefficients and the lowest frequency approximation coefficients, use the inverse high pass filter and inverse low pass filter of the wavelet basis function for upsampling and convolution to obtain the reconstructed signal.
  - Step 2: For each level of detail and approximation coefficients, the inverse high pass filter and inverse low pass filter of the wavelet basis function are used for upsampling and convolution to obtain the reconstructed signal.
  - Step 3: Repeat steps 1 and 2 until all levels of refactoring are completed.
- Output the denoised ECG signals.

##### Normalization

In order to obtain better fitting results and prevent the divergence of training results, it is necessary to standardize the training set, and the standardized equation is:

6 $$\begin{aligned} X = \frac{X - \bar{X}}{\sigma } \end{aligned}$$

Where *X* denotes the training set, $\overline{X}$ denotes the average value of the training set, and $\sigma$ denotes the standard deviation of the training set.

##### Phase space reconstruction

In the model training, we aim to predict the ECG signals in the next time step based on the historical data. It is necessary to use prior data as the training data to predict ECG signals in future time steps. In this paper, the final training set is constructed by phase space reconstruction. If the reconstruction dimension is *m*, the time delay is *tau*, and the ECG signals of the normalized training set is $$X = \left[ \begin{array}{cccc} {x_1},&{x_2},&\cdots ,&{x_{n + 1 + (m - 1)tau}} \end{array}\right]$$, then the reconstructed training set is:

7 $$\begin{aligned} X = \left[ \begin{array}{cccc} {x_1}, &{} {x_2}, &{} \cdots &{} {x_n}\\ {x_{1 + tau}}, &{} {x_{2 + tau}}, &{} \cdots &{} {x_{n + tau}} \\ \vdots &{} \vdots &{} &{} \vdots \\ {x_{1 + (m - 1)tau}}, &{} {x_{2 + (m - 1)tau}} , &{} \cdots &{} {x_{n + (m - 1)tau}} \end{array} \right] \end{aligned}$$

8 $$\begin{aligned} Y = \left[ \begin{array}{ccc} {x_{2 + (m - 1)tau}},&\cdots&{x_{n + 1 + (m - 1)tau}} \end{array}\right] \end{aligned}$$

The ECG signal in the input layer of the predition model are reconstructed from Equations (7) and (8). The construction rule is to start from the first sampling point of the selected ECG signal and use the $1-st$ to $99-th$ sampling points as the input sample, the $100-th$ sample point is taken as the output sample, and so on. The input samples of the $i-th$ training set are the $i~i+98$ sampling points, and the output samples are the $i+99-th$ sampling points, where $i = 1, 2, ..., 5 000-99$. A total of 4901 input-output sample pairs are generated, which is the training data set of the model.

#### Nested LSTMs model

Information exhibits time correlation, and historical information can hold valuable clues for predicting future events. Traditional machine learning methods only have short-term memory, which has prediction limitations in the case of limited information. LSTM introduces ingenious controllable self circulation to generate a path that allows gradient to flow continuously for a long time, which makes it especially suitable for processing tasks related to time series and tracking information in a longer time. As a result, the extended models of LSTM have received increasing attention by virtue of the obvious advantages.

**Fig. 3:**
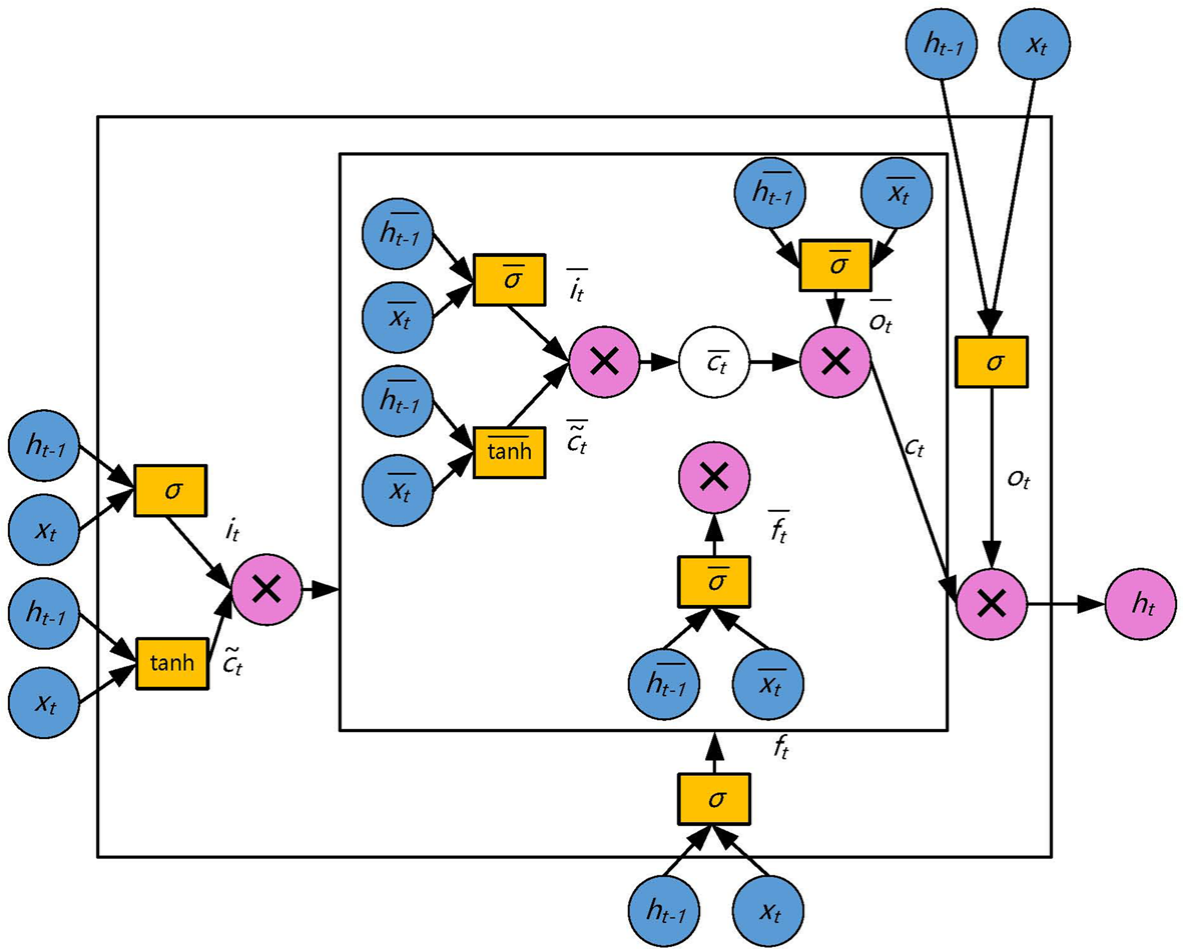
Nested LSTM model unit structure diagram

Nested LSTM shares the same input layer, hidden layer, and output layer as LSTM, and its unit structure is illustrated in Fig. 3. In this figure, a new inner LSTM structure is adopted to replace the memory cells of the traditional LSTM. When accessing the inner memory, they are gated in the same way. Therefore, the Nested LSTM can access the inner memory more specifically, which makes the Nested LSTM prediction model has stronger processing capabilities for ECG signals and higher prediction accuracy. This enhancement allows the Nested LSTM to capture and utilize more intricate temporal patterns in the data, making it well-suited for tasks that require detailed information processing and precise predictions in the context of electrocardiogram signals.

Nested LSTM is divided into inner LSTM and outer LSTM. The gating system of both inner and outer LSTM is consistent with that of traditional LSTM. Within this system, there are four gating systems: forget gate, input gate, candidate memory cell, and output gate. The calculation equations for each gate are as follows.

Forget gate:

9 $$\begin{aligned} {f_t} = \sigma ({W_{fx}}{x_t} + {W_{fh}}{h_{t - 1}} + {b_f}) \end{aligned}$$

Input gate:

10 $$\begin{aligned} {i_t} = \sigma ({W_{ix}}{x_t} + {W_{ih}}{h_{t - 1}} + {b_i}) \end{aligned}$$

Candidate memory cell:

11 $$\begin{aligned} \widetilde{{c_t}} = \tanh ({W_{cx}}{x_t} + {W_{ch}}{h_{t - 1}} + {b_c}) \end{aligned}$$

Memory cell: The input and hidden states of the inner LSTM are:

12 $$\begin{aligned} {\overline{h}_{t - 1}} = {f_t}\ \cdot \ {c_{t - 1}} \end{aligned}$$

13 $$\begin{aligned} {\overline{x}_t} = {i_t}\ \cdot \ \widetilde{{c_t}} \end{aligned}$$

14 $$\begin{aligned} \overline{f}_t = \sigma ({\overline{W}_{fx}}\overline{x_t} + {\overline{W}_{fh}}{\overline{h}_{t - 1}} + {\overline{b}_f}) \end{aligned}$$

15 $$\begin{aligned} \overline{i}_t = \sigma ({\overline{W}_{ix}}\overline{x_t} + {\overline{W}_{ih}}{\overline{h}_{t - 1}}+ {\overline{b}_i}) \end{aligned}$$

16 $$\begin{aligned} \widetilde{\overline{c}}_t = \tanh ({\overline{W}_{cx}}\overline{x_t} + {\overline{W}_{ch}}{\overline{h}_{t - 1}} + {\overline{b}_c}) \end{aligned}$$

17 $$\begin{aligned} \overline{o}_t = \sigma ({\overline{W}_{ox}}\overline{x_t} + {\overline{W}_{oh}}{\overline{h}_{t - 1}} + {\overline{b}_o}) \end{aligned}$$

18 $$\begin{aligned} \overline{c}_t = \overline{f}_t\ \cdot \ {\overline{c}_{t - 1}} + \overline{i}_t{\hspace{1.0pt}} \cdot {\hspace{1.0pt}} \widetilde{\overline{c}}_t \end{aligned}$$

19 $$\begin{aligned} \overline{h} _t= \overline{o}_t\ \cdot \tanh (\overline{c}_t) \end{aligned}$$

The memory cell update method of the outer LSTM is:

20 $$\begin{aligned} {c_t} = \overline{{h}}_t \end{aligned}$$

Output gate:

21 $$\begin{aligned} {o_t} = \sigma ({W_{ox}}{x_t} + {W_{oh}}{h_{t - 1}} + {b_o}) \end{aligned}$$

A new round of hidden state:

22 $$\begin{aligned} {h_t} = {o_t}\ \cdot \ \tanh ({c_t}) \end{aligned}$$

Where $\sigma$ denotes the sigmoid function. In the outer LSTM, ${W_{fx}}$ and ${W_{fh}}$ denote the weight matrix of the forget gate; ${W_{ix}}$ and ${W_{ih}}$ denote the weight matrix of the input gate; ${W_{cx}}$ and ${W_{ch}}$ denote the weight matrix of the candidate memory cell; ${W_{ox}}$ and ${W_{oh}}$ denote the weight matrix of the output gate; ${b_f}$, ${b_i}$, ${b_c}$ and ${b_o}$ denote the bias of the forget gate, input gate, candidate memory cell, and output gate respectively. In the inner LSTM, $\overline{x}_t$, $\overline{h}_{t - 1}$ and $\overline{c}_{t - 1}$ denote the current input, the hidden state and memory cell of the previous round, respectively. In the outer LSTM, ${\overline{W}_{fx}}$ and ${\overline{W}_{fh}}$ denote the weight matrix of the forget gate; ${\overline{W}_{ix}}$ and ${\overline{W}_{ih}}$ denote the weight matrix of the input gate; ${\overline{W}_{cx}}$ and ${\overline{W}_{ch}}$ denote the weight matrix of the candidate memory cell; ${\overline{W}_{ox}}$ and ${\overline{W}_{oh}}$ denote the weight matrix of the output gate; $\overline{b}_f$, $\overline{b}_i$, $\overline{b}_c$ and $\overline{b}_o$ denote the bias of the forget gate, input gate, candidate memory cell, and output gate respectively; $\overline{x}_t$, $\overline{h}_{t - 1}$ and $\overline{c}_{t - 1}$ denote the current input, the hidden state and memory cell of the previous round, respectively.

The output of the output layer is:

23 $$\begin{aligned} {y_t} = \sigma ({W_{yh}}{h_t}) \end{aligned}$$

Where ${W_{yh}}$ denotes the weight matrix of the output layer.

The Nested LSTM model is a deep neural network that incorporates both feedforward and feedback mechanisms. The feedforward mechanism completes the forward calculation of the Nested LSTM using equations (6)-(20). In contrast, the feedback mechanism employs the error backpropagation algorithm to train and update various network parameters.

The training process of the feedback mechanism first needs to define the loss function:

24 $$\begin{aligned} {E_t}= & {} \frac{1}{2}{({y_t} - \overset{\wedge }{y_t}\ )^2}\nonumber \\ E= & {} \sum \limits _{t = 1}^T {{E_i}} \end{aligned}$$

Where ${E_t}$ denotes the error at time *t*, *E* denotes the total error, ${y_t}$ denotes the training value, and $\overset{\wedge }{y_t}$ is the target value.

**Fig. 4:**
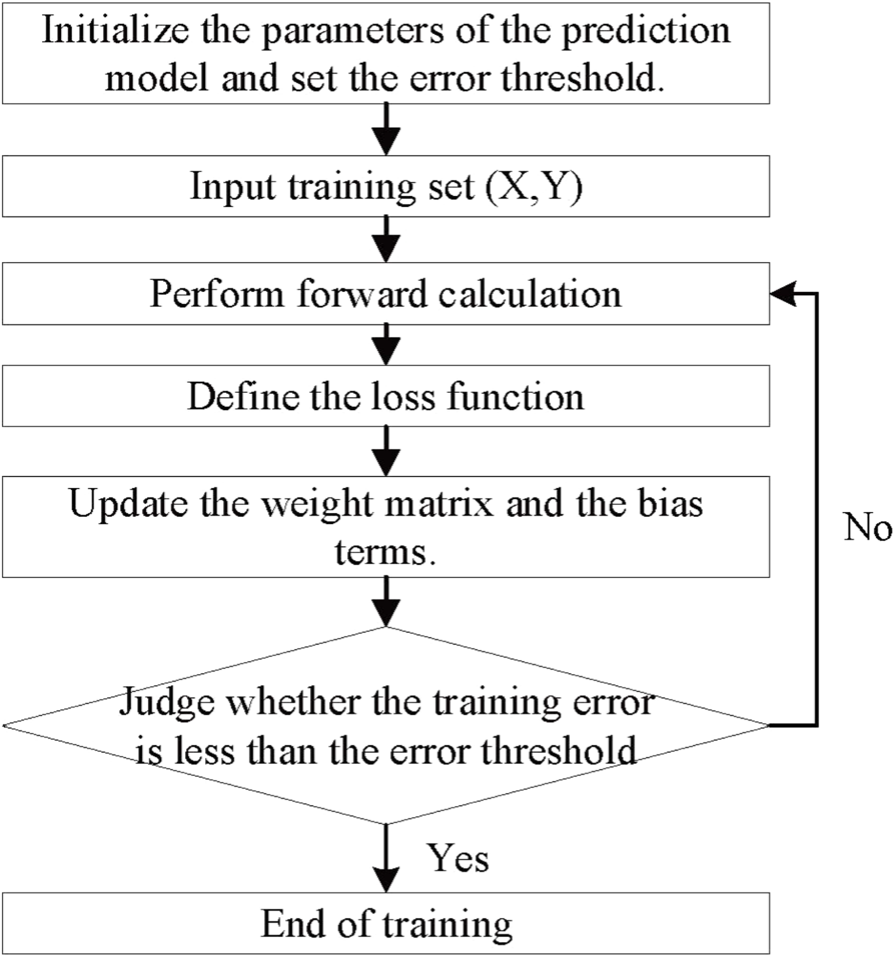
Training flowchart of prediction model

The weight matrix and the bias term of each gating system need to be updated by the loss function [31]. Its process is shown in Fig. 4 and the specific steps are as follows:

- Initialize the parameters of the prediction model and set the error threshold.
- Input the ECG signals training set.
- Perform forward calculation according to equations (6)-(20) to obtain the output corresponding to the current input.
- Define the loss function.
- Solve the gradient of each weight according to the loss function, and then update the weight matrix according to the gradient guide and update the bias terms.
- Judge whether the training error is less than the error threshold; if yes, skip to Step7; if not, skip to Step3.
- End of training.

## Experiment and results

### Data source

In this study, ECG signals were obtained from the Sudden Cardiac Death Holter Database on the PhysioNet website [37], a resource for complex physiological and biomedical signal research. The database features 20 patient groups who experienced actual cardiac arrest and exhibited potential sinus rhythm, persistent rhythm, and atrial fibrillation prior to the event. Medical experts have meticulously annotated these ECG signals, identifying the onset of sudden cardiac death. The dataset for this study consists of 20 sets of 20 second predicted signals (20 seconds including 16 seconds before and 4 seconds after the SCD event). The detailed information of 20 groups ECG signal used in this paper are shown in Table 1. We randomly select a set of ECG signals as the training set, that is, randomly create a model for each subject. The construction rule is to start from the first sampling point of the selected ECG signal and use the $1_{st}$ to $99_{th}$ sampling points as the input sample, the $100_{th}$ sample point is taken as the output sample, and so on. The input samples of the $i_{th}$ training set are the $i-i+98$ sampling points, and the output samples are the $i+99th$ sampling points, where $i = 1, 2, ..., 5 000-99$. A total of 4901 input-output sample pairs are generated, which is the training data set of the model.

**Table 1:** ECG Signals Information

Signals (.dat)	Gender	Age	Signal Duration	VF Onset Time (elapsed)
30	Male	43	24:33:17	07:54:33
31	Female	72	13:58:40	13:42:24
32	Unknown	62	24:20:00	16:45:18
33	Female	30	24:33:00	04:46:19
34	Male	34	07:05:20	06:35:44
35	Female	72	24:52:00	24:34:56
36	Male	75	20:21:20	18:59:01
37	Female	89	25:08:00	01:31:13
38	Unknown	Unknown	18:18:25	08:01:54
39	Male	66	05:47:00	04:37:51
41	Male	Unknown	03:56:00	02:59:24
43	Male	35	23:07:50	15:37:11
44	Male	Unknown	23:20:00	19:38:45
45	Male	68	24:09:20	18:09:17
46	Female	Unknown	04:15:10	03:41:47
47	Male	34	23:35:50	06:13:01
48	Male	80	24:36:15	02:29:40
50	Female	68	23:07:38	11:45:43
51	Female	67	25:08:30	22:58:23
52	Female	82	07:31:05	02:32:40

### Data preprocessing results

**Fig. 5:**
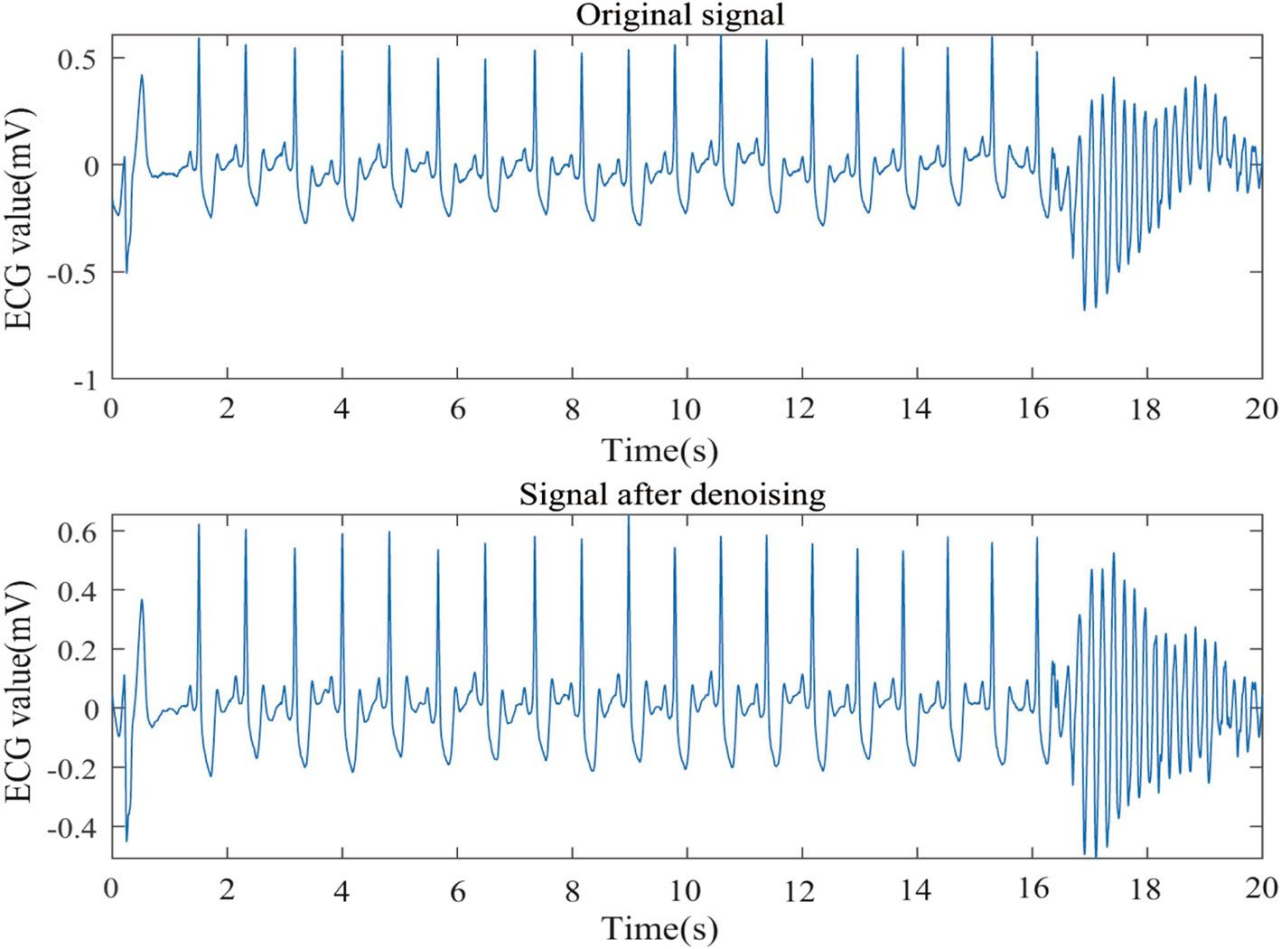
Comparison of 30.dat signal before and after denoising

**Fig. 6:**
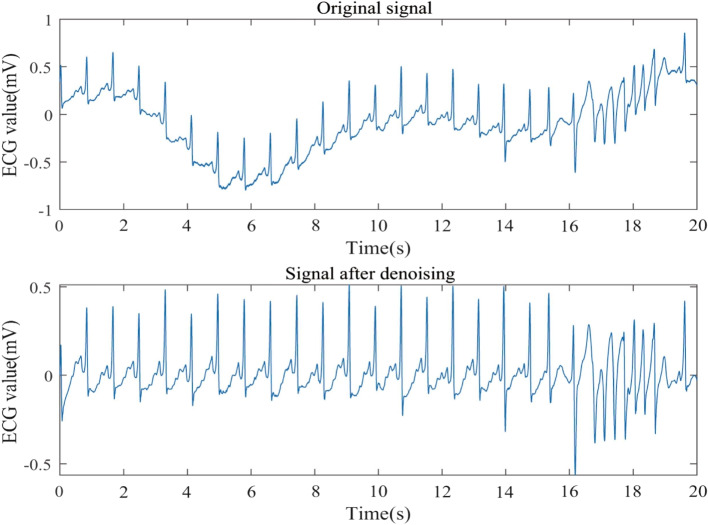
Comparison of 31.dat signal before and after denoising

**Fig. 7:**
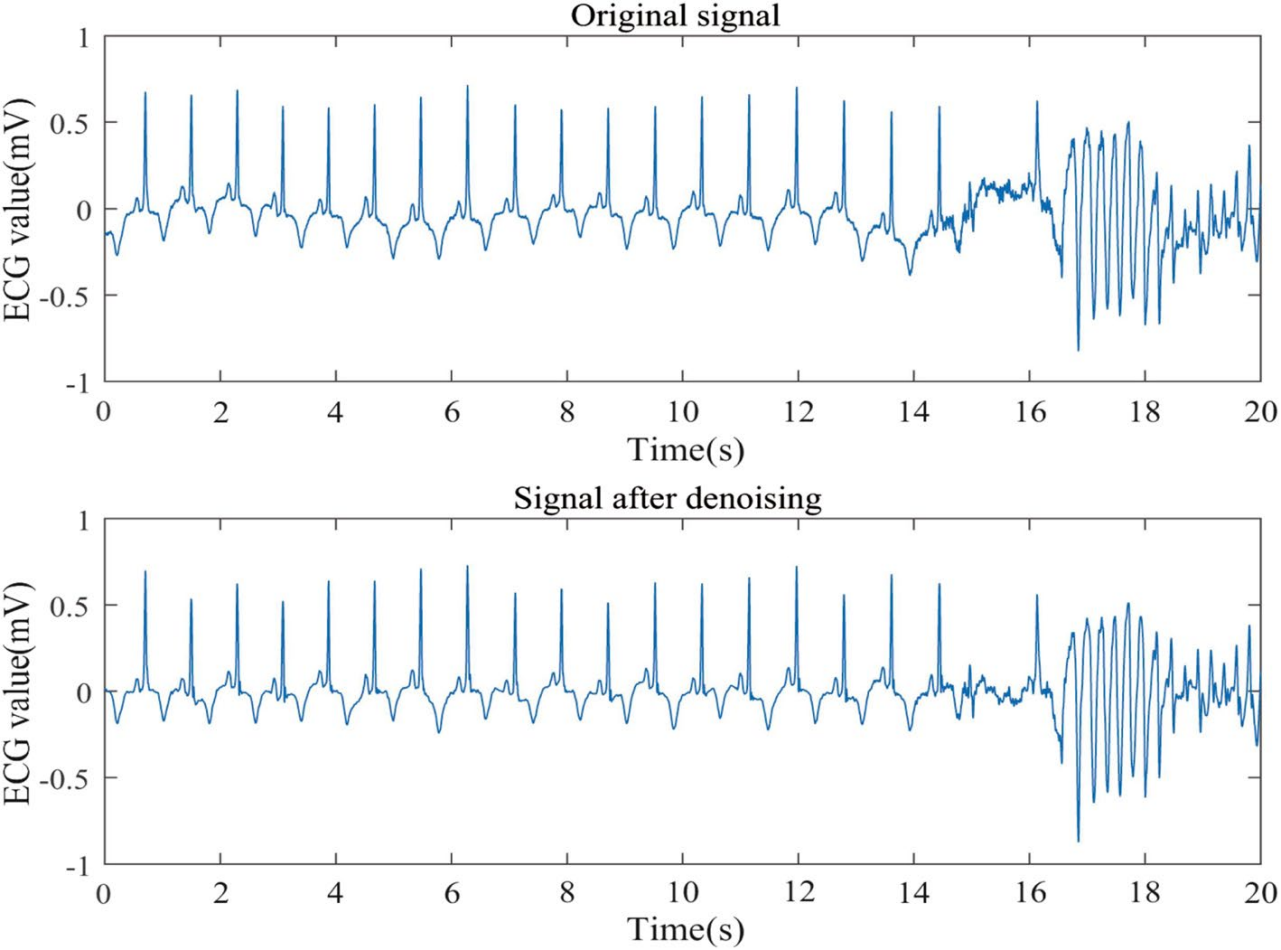
Comparison of 32.dat signal before and after denoising

Utilizing all ECG signals as model input may introduce noise and decrease prediction accuracy. As a result, a wavelet denoising method is employed to remove noise. Denoising outcomes are displayed in Figs. 5, 6 and 7.

From Figs. 5, 6 and 7, it is evident that the signals 30.dat and 31.dat exhibit noticeable baseline drift noise before denoising, while signal 32.dat exhibits both baseline drift and EMG noise. However, after denoising, the signals 30.dat-32.dat become more stable (as supported by Table 2). This demonstrates that the enhanced wavelet denoising method employed in this study effectively eliminates baseline drift and EMG signal noise, indicating its capability to denoise ECG signals.

**Table 2:** SNR results

Signals (.dat)	Before denoising (dB)	After denoising (dB)
30	-18.68	12.33
31	-22.13	10.82
32	-16.55	13.19

This study assesses the denoising effect through visual and signal-to-noise ratio (SNR). The denoising results are further compared using SNR, a technical metric that measures the ratio of signal energy to noise energy. The specific definitions are as follows:

25 $$\begin{aligned} SNR = 10\log \frac{{\sum \limits _{n = 1}^N {{{[x(n)]}^2}} }}{{\sum \limits _{n = 1}^N {{{[{x_m}(n) - x(n)]}^2}} }} \end{aligned}$$

Where *x*(*n*) denotes the original signal, and ${x_m}(n)$ denotes the denoising signal. We can evaluate the denoising effect by comparing the SNR of ECG signal before and after denoising. The SNR results are shown in Table 2.

Table 2 demonstrates that a portion of the noise in the aforementioned ECG signals has been removed, as evidenced by the increased SNR before and after denoising. This finding more clearly indicates the presence of a significant amount of noise in the ECG signal, which could impact prediction outcomes. Consequently, it is necessary to employ a denoising method to process the ECG signal prior to making predictions.

### Predicion results and analysis

**Fig. 8:**
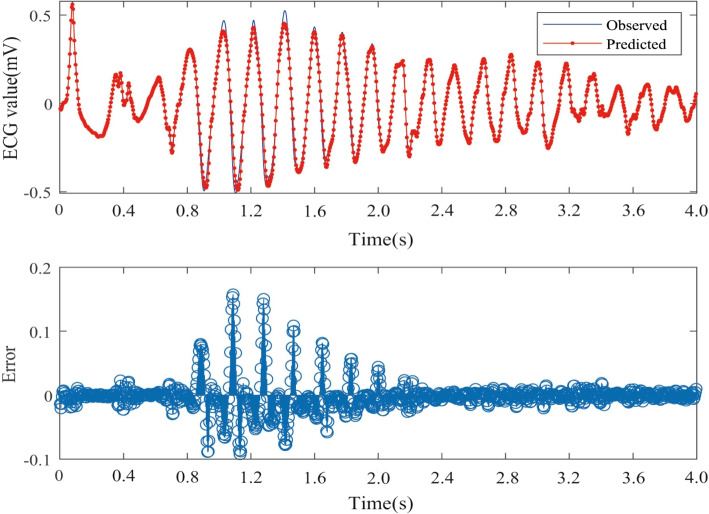
One-step prediction fitting result and error result of 30.dat signal

**Fig. 9:**
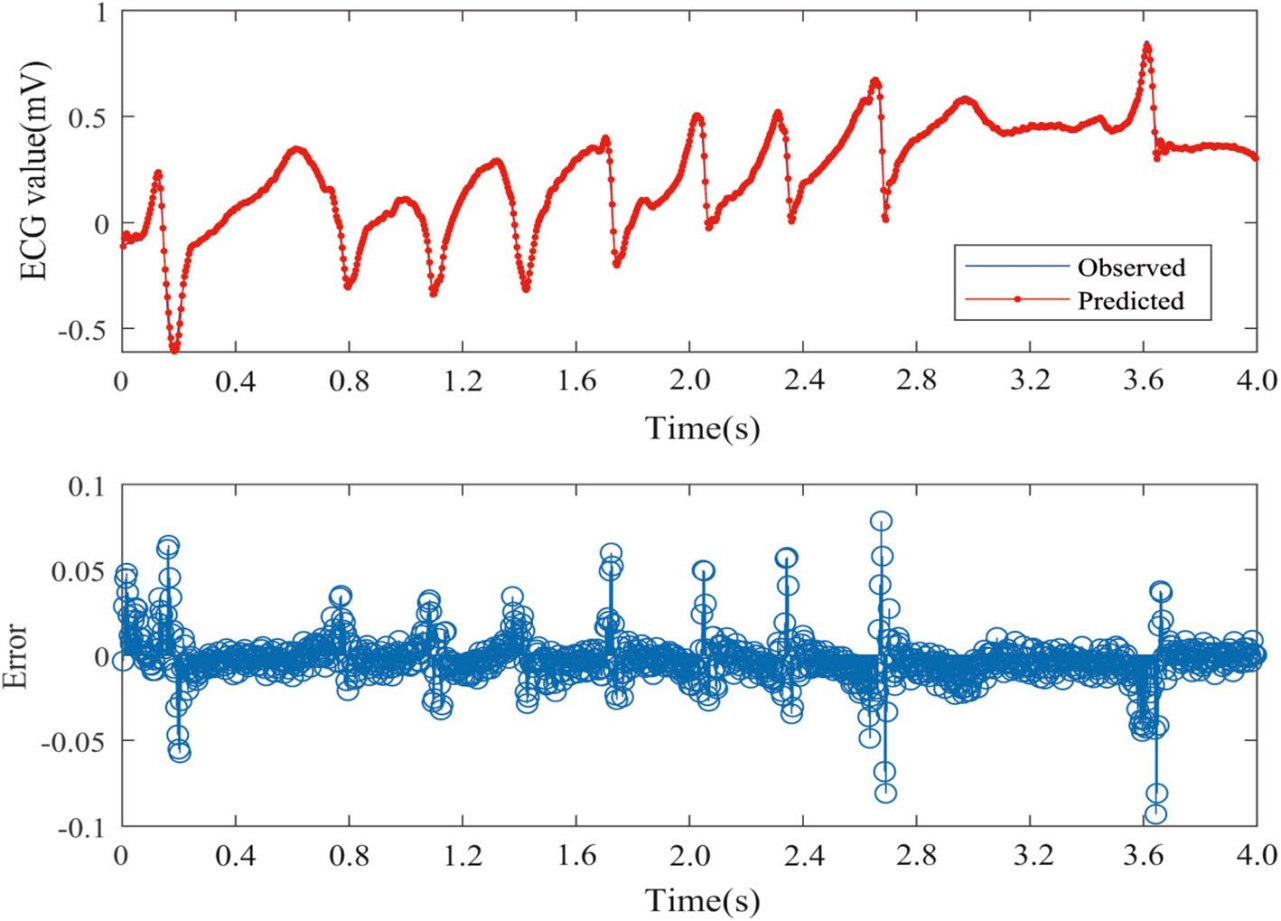
One-step prediction fitting result and error result of 31.dat signal

**Fig. 10:**
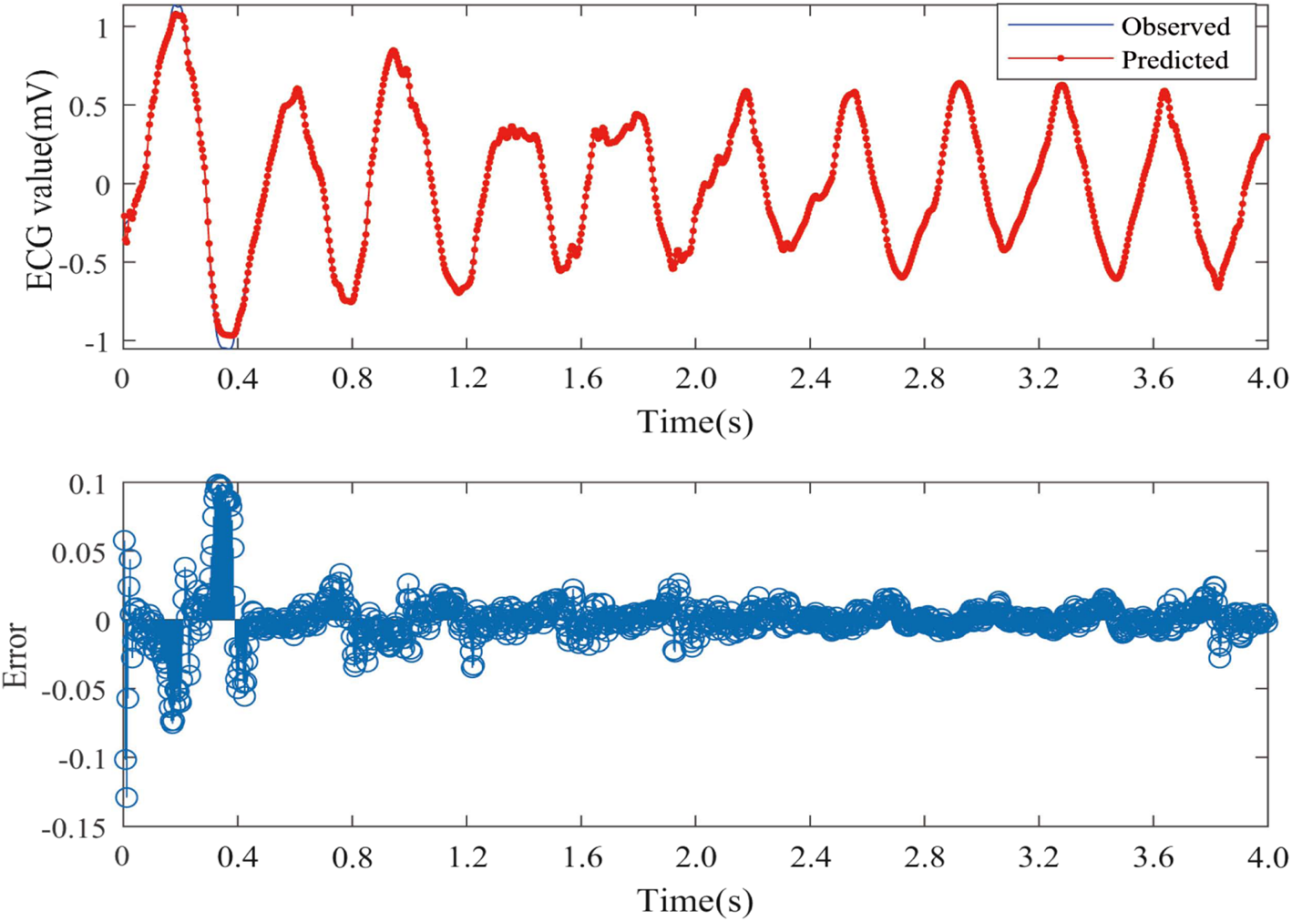
One-step prediction fitting result and error result of 32.dat signal

The Nested LSTM model was used to predict the risk of actual cardiac arrest for the 20 groups of ECG signals. In the experiment, we trained the model 20 times in total, and the training time was between 43s-58s. A selection of prediction results is presented in Figs. 8, 9 and 10. The error is calculated as the difference between the true value and the predicted value.

Figures 8, 9, and 10 illustrate notable fluctuations in the ECG signal. The actual values are represented in blue, while the fitted predictions are shown in red. Impressively, the proposed method exhibits remarkable proficiency in capturing these variations and accurately predicting trends in the ECG signal. The experimental results highlight the potential of predicting sudden cardiac death (SCD) before its occurrence, which holds life-saving implications for patients.

There are several techniques for classifying the risk of SCD. Support Vector Machines (SVM) is a classical classification method that is widely used. Echo State Networks (ESN) and Long Short-Term Memory (LSTM) networks are adaptive data analysis methods that have been employed in SCD detection. Bidirectional LSTM (Bi-LSTM) is an improved method of LSTM, comprising forward and backward LSTM components, which enables the summarization of temporal information from both past and future contexts. To validate the performance of the Nested LSTM model, the four models mentioned above are compared. In order to quantitatively assess the performance of the predictive models, two types of error measurements are employed in the experiment: mean absolute error (MAE) and root mean square error (RMSE).

26 $$\begin{aligned} RMSE = \sqrt{\frac{1}{N}{\sum \limits _{n = 1}^N ({Y_n} - \widehat{Y_n} )^2}} \end{aligned}$$

27 $$\begin{aligned} MAE = \frac{1}{N}\sum \limits _{n = 1}^N {\left| {{Y_n} - \widehat{Y_n} } \right| } \end{aligned}$$

**Fig. 11:**
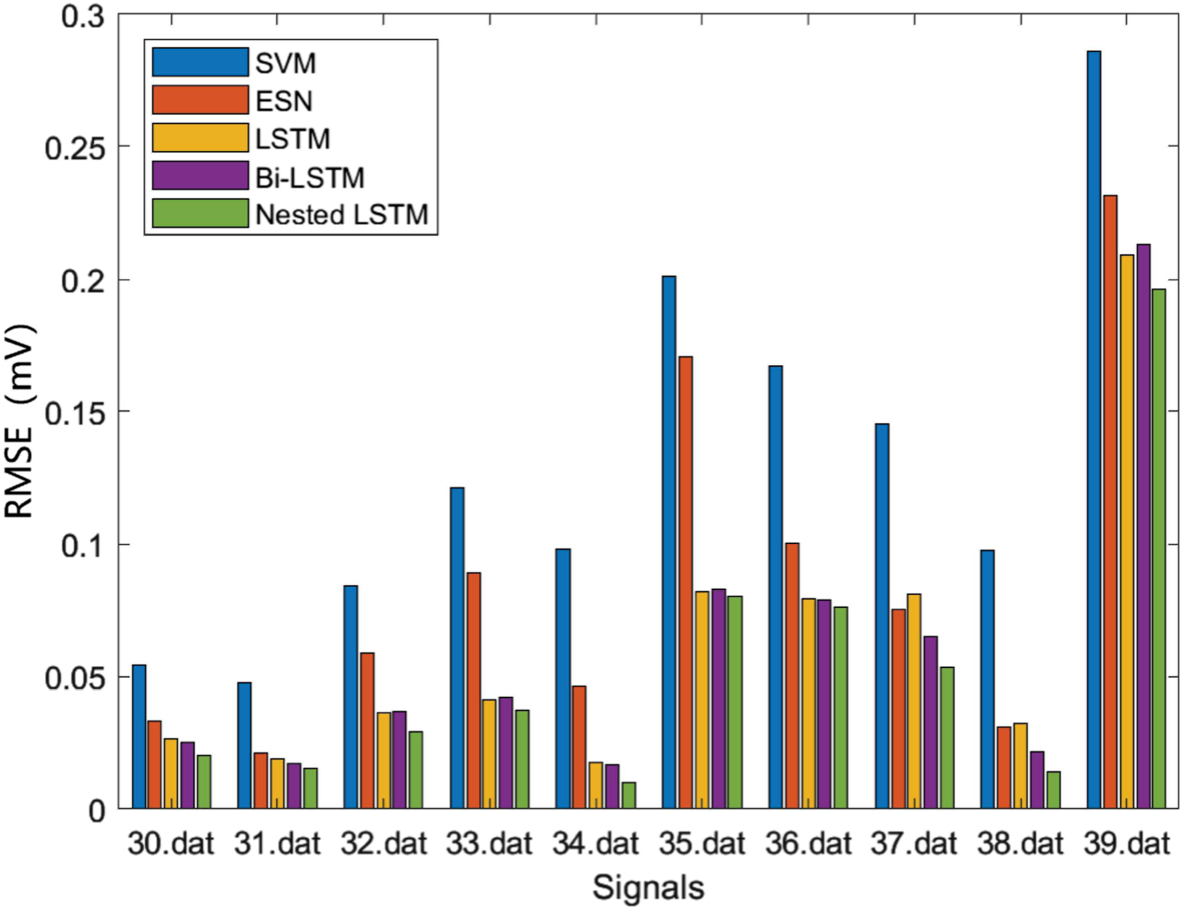
RMSE results (a)

**Fig. 12:**
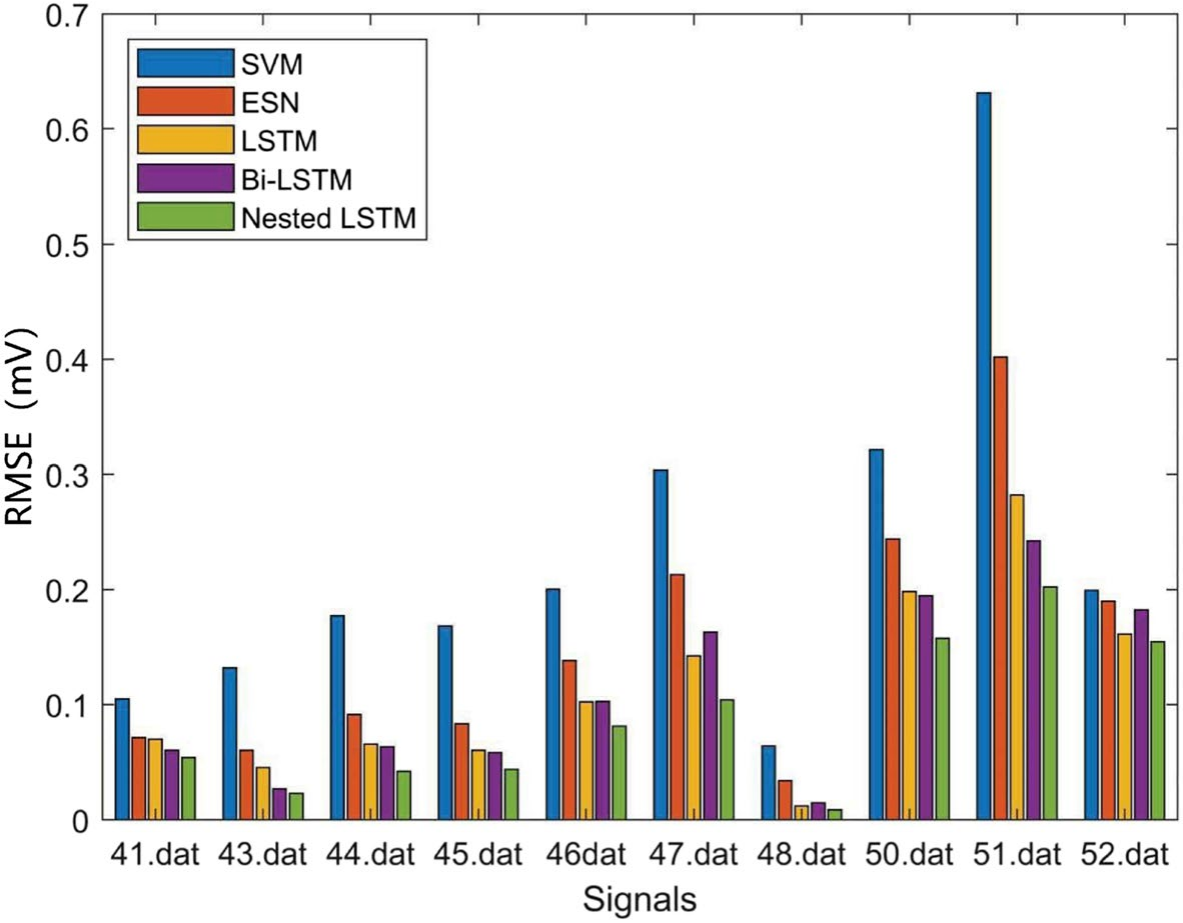
RMSE results (b)

**Fig. 13:**
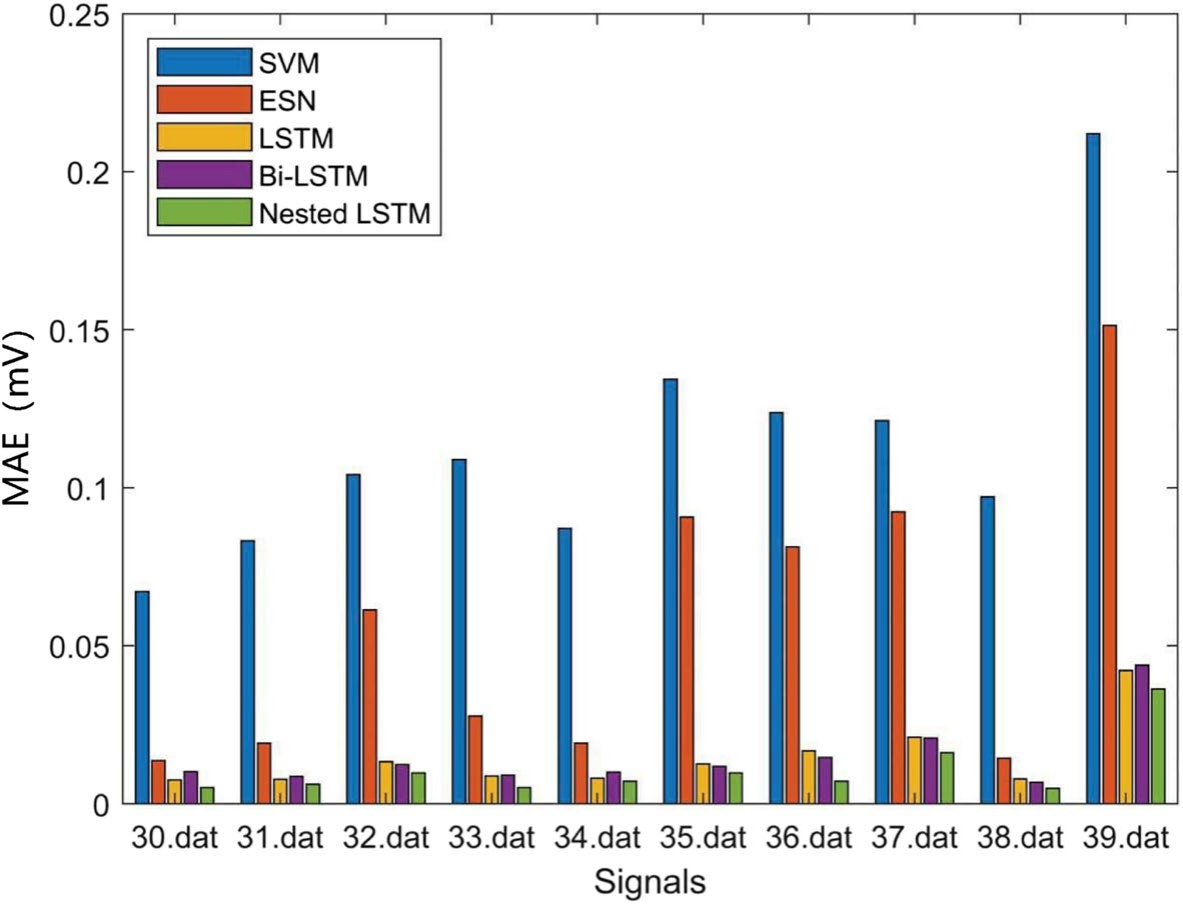
MAE results (a)

**Fig. 14:**
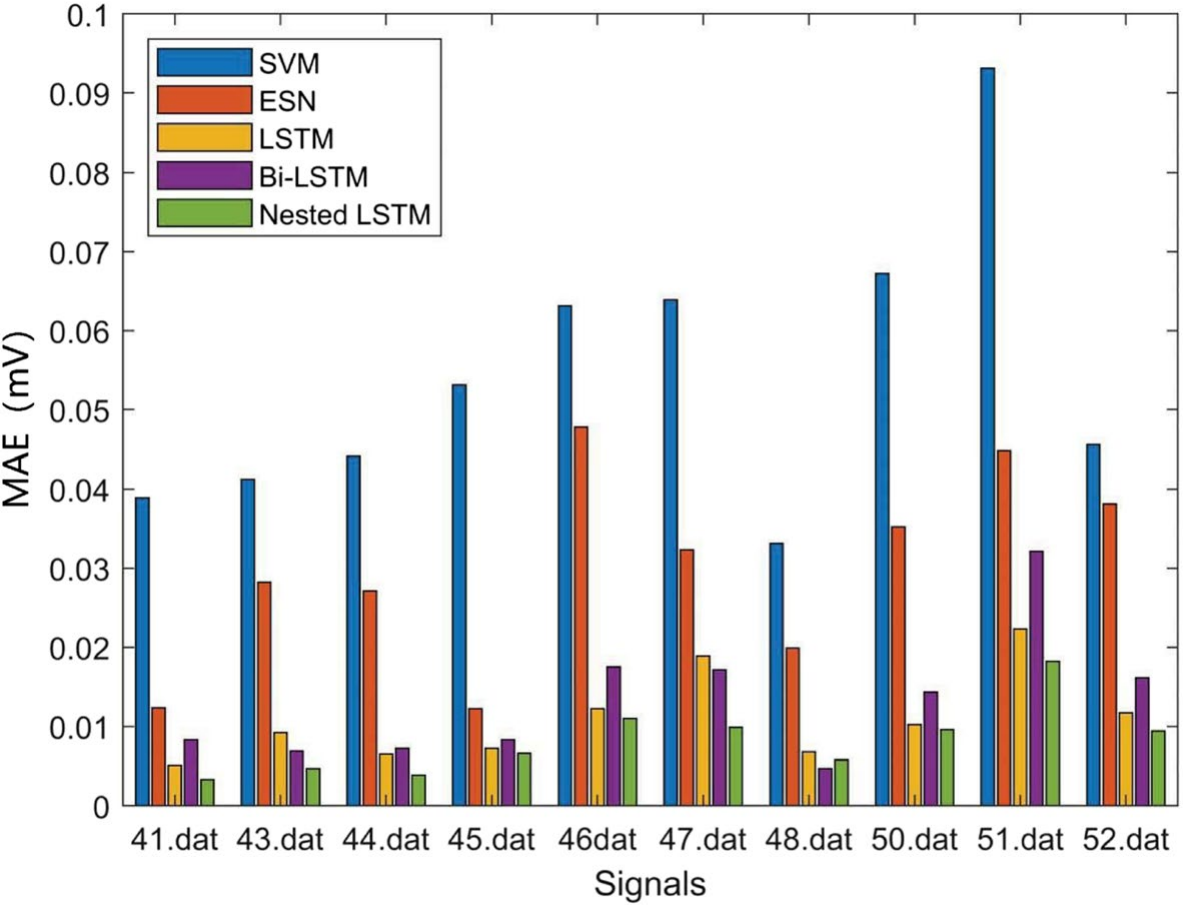
MAE results (b)

Where *N* denotes the predicted ECG signals length, ${Y_n}$ denotes the predicted value, and $\widehat{Y_n}$ denotes the target value. The prediction error results are averaged. The prediction RMSE results of the above models are shown in Table 3, Figs. 11 and 12, the prediction MAE results of the above models are shown in Table 4, Figs. 13 and 14, and the prediction average results of the above models are shown in Table 5.

**Table 3:** RMSE results

Signals(.dat)	SVM(mV)	ESN(mV)	LSTM(mV)	Bi-LSTM(mV)	Nested LSTM(mV)
30	0.0542	0.0332	0.0267	0.0254	0.0201
31	0.0477	0.0212	0.0188	0.0172	0.0152
32	0.0841	0.0589	0.0364	0.0369	0.0292
33	0.1211	0.0889	0.0415	0.0421	0.0372
34	0.0982	0.0463	0.0177	0.0168	0.0102
35	0.2011	0.1706	0.0821	0.0827	0.0801
36	0.1671	0.1001	0.0792	0.0789	0.0762
37	0.1452	0.0753	0.0812	0.0649	0.0532
38	0.0977	0.0310	0.0322	0.0218	0.0141
39	0.2860	0.2314	0.2091	0.2133	0.1964
41	0.1048	0.0711	0.0699	0.0603	0.0539
43	0.1320	0.0603	0.0451	0.0271	0.0231
44	0.1773	0.0912	0.0655	0.0632	0.0419
45	0.1682	0.0831	0.0602	0.0582	0.0436
46	0.2003	0.1382	0.1021	0.1025	0.0811
47	0.3039	0.2129	0.1421	0.1628	0.1039
48	0.0639	0.0342	0.0121	0.0147	0.0088
50	0.3211	0.2440	0.1982	0.1945	0.1576
51	0.6311	0.4018	0.2821	0.2421	0.2022
52	0.1993	0.1899	0.1612	0.1821	0.1544

**Table 4:** MAE results

Signals(.dat)	SVM(mV)	ESN(mV)	LSTM(mV)	Bi-LSTM(mV)	Nested LSTM(mV)
30	0.0671	0.0138	0.0076	0.0102	0.0052
31	0.0832	0.0192	0.0078	0.0087	0.0063
32	0.1041	0.0612	0.0134	0.0125	0.0098
33	0.1089	0.0278	0.0089	0.0091	0.0052
34	0.0871	0.0192	0.0081	0.0101	0.0072
35	0.1344	0.0906	0.0127	0.0118	0.0098
36	0.1237	0.0812	0.0169	0.0147	0.0072
37	0.1212	0.0923	0.0211	0.0209	0.0162
38	0.0971	0.0145	0.0079	0.0068	0.0049
39	0.2120	0.1514	0.0421	0.0437	0.0364
41	0.0389	0.0123	0.0051	0.0083	0.0033
43	0.0412	0.0282	0.0092	0.0069	0.0047
44	0.0441	0.0271	0.0065	0.0072	0.0039
45	0.0531	0.0122	0.0072	0.0083	0.0066
46	0.0631	0.0478	0.0122	0.0175	0.0110
47	0.0639	0.0323	0.0189	0.0171	0.0099
48	0.0331	0.0199	0.0068	0.0047	0.0058
50	0.0672	0.0352	0.0102	0.0143	0.0096
51	0.0931	0.0448	0.0223	0.0321	0.0182
52	0.0456	0.0381	0.0117	0.0161	0.0094

**Table 5:** Average results

Signals	SVM(mV)	ESN(mV)	LSTM(mV)	Bi-LSTM(mV)	Nested LSTM(mV)
Average RMSE	0.1802	0.1192	0.0882	0.0854	0.0701
Average MAE	0.0841	0.0436	0.0128	0.0141	0.0095

RMSE can reflect the overall error of signal prediction. According to Figs. 11, 12 and Table 3, it can be observed that the SVM model has the highest RMSE. The SVM model, which lacks a feedback structure and cannot obtain historical information characteristics, performs poorly in ECG signal prediction. The ESN model, despite having a feedback structure, is simple and also exhibits a large error. The LSTM and Bi-LSTM models have low RMSE values, with their results being quite similar. Nested LSTM enhances the memory function of LSTM and extracts historical information features more accurately. When compared to the SVM, ESN, LSTM, and Bi-LSTM models, the RMSE of Nested LSTM is the smallest.

MAE represents the average of the absolute error between the predicted value and the observed value. According to Figs. 13, 14 and Table 4, it can be seen that the SVM model also has the largest MAE, and the Nested LSTM has the smallest MAE among the five models.

According to Table 5, it can be seen that the SVM model has the largest average RMSE and average MAE, and the Nested LSTM model has the smallest average RMSE and average MAE. When compared to the SVM, ESN, LSTM, and Bi-LSTM models, the average RMSE of Nested LSTM is reduced by 61.1%, 41.2%, 20.5%, and 17.9%, respectively. And the average MAE of Nested LSTM is reduced by 88.7%, 78.2%, 25.8%, and 32.6%, respectively. In conclusion, the Nested LSTM model demonstrates a strong nonlinear mapping ability for ECG signals.

## Conclusion

In this study, we present an early prediction method for sudden cardiac death (SCD) risk using Nested LSTM based on electrocardiogram (ECG) sequential features to predict a patient’s ECG signals. ECG prediction is an effective approach for the early prediction of SCD risk. One limitation of the traditional prediction methods is that it has a low predict accuracy for strong nonlinear ECG, which may turn out to be inappropriate for practical applications. Thus, it is highly desirable to develop an optimized ECG prediction model with a high prediction accuracy. Focusing on the timeliness and accuracy of prediction, this paper focuses on the nonlinear mapping capability of Nested LSTM for ECG signals. The memory cell of Nested LSTM is replaced by an inner LSTM, which has strong memory ability. To demonstrate the effectiveness and applicability of the proposed model, the ECG signals of 20 groups of actual cardiac arrest patients are taken for conducting the empirical study. The experiment results show that the proposed model achieves better performance in comparison with other four models.

The current similar methods include classification and regression techniques. References [14–16] employ classification models to anticipate abnormal and non-abnormal ECG patterns, whereas references [21–23] utilize regression models to align with ECG signal trends and identify ECG outliers. Distinguished from conventional classification techniques, this approach excels in forecasting the onset of SCD heartbeats, effectively capturing the dynamic, nonlinear, and non-stationary nature of time series, and adeptly accommodating the irregular trends in electrocardiogram signals. Furthermore, in contrast to traditional regression methodologies, the study devises an encompassing strategy that merges data preprocessing with predictive model development for ECG prediction. Empirical findings demonstrate a notable reduction in fitting errors, specifically in terms of RMSE and MAE, underscoring the efficacy of this novel methodology.

As for future work, we will study the multi-step prediction method of ECG signal characteristics, and use a series of deep learning methods and reinforcement learning methods to reduce multi-step prediction errors. More significantly, the practical applicability of ECG signal prediction methods will be verified in SCD diagnostic applications, potentially saving patients’ lives from SCD events.

## Data Availability

The datasets used and/or analysed during the current study available from the corresponding author on reasonable request.
